# Prevalence of Disease

**Published:** 1856-03

**Authors:** 


*Prevalence of Disease.*

From many of our correspondents in different sections of

Illinois, Indiana, and Iowa, we learn that pneumonia is quite prevalent in their respective neighborhoods. This is what we should expect on the decline of a winter so protracted and intensely cold as that of 1855–6. Several of the correspondents also mention the coincident prevalence of Typhoid Fever to an unusual degree. Dr. J. N. Higgins, of Griggsville, in this State, writes: "We have had considerable pneumonia in this vicinity recently, and in many cases where the fever was pretty active, I have used the tinct. veratrum viride with perfect success. Also, in a severe case of inflammatory rheumatism, when the cimicifuga was not at hand, an administration of the tinct. of veratrum, until nausea was produced, at once checked the disease. I use a tincture prepared by J. H. Reed & Co., of Chicago, which I consider fully equal to Norwood's, and at less than half the price. In several instances, I have commenced with drop doses, increasing one drop each dose, until nausea was produced. In no instance have I been disappointed in its controlling influence upon the heart's action, as well as in its diaphoretic power." Other practitioners speak equally favorable concerning the value of the veratrum in the treatment of active, febrile and inflammatory affections. In this city, during the last half of February and the first week in March, attacks of pneumonia were unusually frequent in *young* children.

We have noticed, also, during the same period of time, an unusual number of attacks of sub-acute meningitis, in children under two years of age; and in several instances the pneumonic and cerebral inflammations existed co-incidently in the same cases. During the first week in March, we met with two or three well marked and severe cases of pernicious or malignant intermittents.

